# Association of healthy lifestyle and physical fitness in children: a nationwide population-based cross-sectional study

**DOI:** 10.3389/fpubh.2026.1885065

**Published:** 2026-07-08

**Authors:** Mengmeng Fan, Yuan Liu, Jie Shen, Changzheng Yuan, Xiaojian Yin, Min Hou

**Affiliations:** 1 School of Public Health, Shanghai Jiao Tong University College of Medicine, Shanghai, China; 2 School of Physical Education, Shanghai University, Shanghai, China; 3 School of Public Health, The Second Affiliated Hospital, Zhejiang University School of Medicine, Hangzhou, China; 4 Department of Physical Education, Shanghai Institute of Technology, Shanghai, China

**Keywords:** child, diet, life style, physical fitness, screen time

## Abstract

**Objective:**

Although a healthy lifestyle is closely linked to various health outcomes in children and adolescents, its specific connection to physical fitness remains poorly understood.

**Methods:**

This nationwide cross-sectional study evaluated data from 60,177 children across 27 provinces in China from 2015 to 2016. Health Lifestyle Score (HLS) assessed four key lifestyle factors: healthy eating, sufficient sleep, regular physical activity, and limited screen time. Multiple linear regression models and restricted cubic spline assessed the association between HLS and general physical fitness, with individual lifestyle factors also examined independently.

**Results:**

Higher HLS was significantly associated with better general physical fitness. Positive correlations were observed for each lifestyle factor with physical fitness: healthy eating (*β*, 0.49; 95% CI, 0.39 to 0.59), sufficient sleep (*β*, 0.12; 95% CI, 0.04 to 0.20), regular physical activity (*β*, 0.40; 95% CI, 0.34 to 0.46) and low screen time (*β*, 1.34; 95% CI, 1.24 to 1.45). Limited screen time showed the strongest association, followed by healthy eating. Improvements in the Physical Fitness Index and individual adaptability climbed steadily with each added healthy lifestyle factor.

**Conclusion:**

Future strategies should integrate screen time reduction, dietary improvements, increased physical activity, and adequate sleep to maximize health benefits in this population.

## 1 Introduction

Physical fitness plays a critical role in the mental, social and psychological well-being of children and adolescents (1), which include cardiorespiratory fitness (CRF), muscular strength and endurance, flexibility, agility, coordination, and explosive power (2). Among these, higher CRF have been linked to greater hippocampal volume and basal ganglia development (3), which are associated with enhanced brain function. Additionally, physical fitness, particularly lower limb strength and CRF, has been shown to promote social competence (4). Furthermore, physical fitness is positively associated with academic performance, with greater perseverance and higher fitness levels linked to enhanced intelligence and improved academic outcomes (5). These findings underscore the importance of promoting physical fitness for optimal growth and development in youth.

It is suggested that lifestyle behaviors often interconnected, should be analyzed collectively to better evaluate their influence on health outcomes (6). A healthy lifestyle, comprising healthy diet, regular physical activity and adequate sleep, is known to influence life expectancy and chronic diseases risk, including cardiovascular diseases and obesity (7). However, most studies examining lifestyle factors and physical fitness have focused on individual behaviors, often with small sample sizes and limited generalizability. For example, greater adherence to the Mediterranean diet has been associated with better physical fitness, particularly among boys (8), while interventions targeting physical activity have led to significant improvements in VO2max (9). Similarly, systematic review suggest that longer and higher-quality sleep is linked to improved physical fitness outcomes (10). Notably, one study involving 189 adolescents assessed the impact of a comprehensive healthy lifestyle index, including physical activity, sedentary time, diet, and sleep, on CRF. The findings revealed that adolescents with higher healthy lifestyle index scores exhibited better CRF over 24 months, compared to those with lower scores (11).

The widespread screen use presents an additional challenge for children and adolescents. Excessive screen time has been linked to negative physical, psychosocial, and cognitive outcomes in children (12). A study of 8,136 Chinese adolescents found a significant association between prolonged screen time and poorer physical fitness outcomes (13). Despite these findings, the extent to which a comprehensive set of lifestyle factors, including diet, physical activity, sleep, and screen time, jointly influence general and multidimensional aspects of physical fitness remains unclear. To address this gap, we conduct a large-scale nationwide cross-sectional study, developing a composite Healthy Lifestyle Score (HLS) to evaluate adherence to key lifestyle behaviors. Additionally, we assessed seven standardized fitness tests to capture multiple fitness domains. This study aims to provide robust evidence on the combined association between healthy lifestyle and physical fitness in children and adolescent, offering insights for future health promotion strategies.

## 2 Materials and methods

### 2.1 Data source and collection procedure

Data for this study were drawn from the “Preparation of New Evaluation Methods and Criteria for Physical Health of Children and Adolescents in China” (No. 11001-412,221-15017). It was approved by the Human Experimental Ethics Committee of the East China Normal University (Approval No.: HR2016/12055) and was conducted between May 2015 and May 2016 by the Key Laboratory of Adolescent Health Assessment and Exercise Intervention of the Ministry of Education. The 2015 and 2016 survey waves were conducted as independent cross-sectional surveys, with no participant overlap between survey years. Its primary purpose is to monitor the growth, development, and health status of children and adolescents across China.

Data collection was organized and carried out by provincial and municipal teams of trained examiners (including school doctors, physical education teachers, and local health workers) following a standardized national protocol. All examiners underwent centralized training to ensure consistency in measurement techniques and procedures.

### 2.2 Study design and population

A stratified random cluster sampling design was employed to cover 27 provinces, ensuring broad geographical and socioeconomic representation. All students included in the study met the following criteria: (1) currently enrolled in school; (2) aged between 7 and 18 years; (3) without physical or intellectual disabilities that could impede their participation; and (4) provided voluntary consent to participate. Written informed consent was obtained from all participants and their guardians. In addition, verbal assent was obtained from all child participants prior to any testing or interviews. This study was conducted in accordance with the Enhanced Observational Epidemiological Study Reports (STROBE) guidelines.

**Figure 1:**
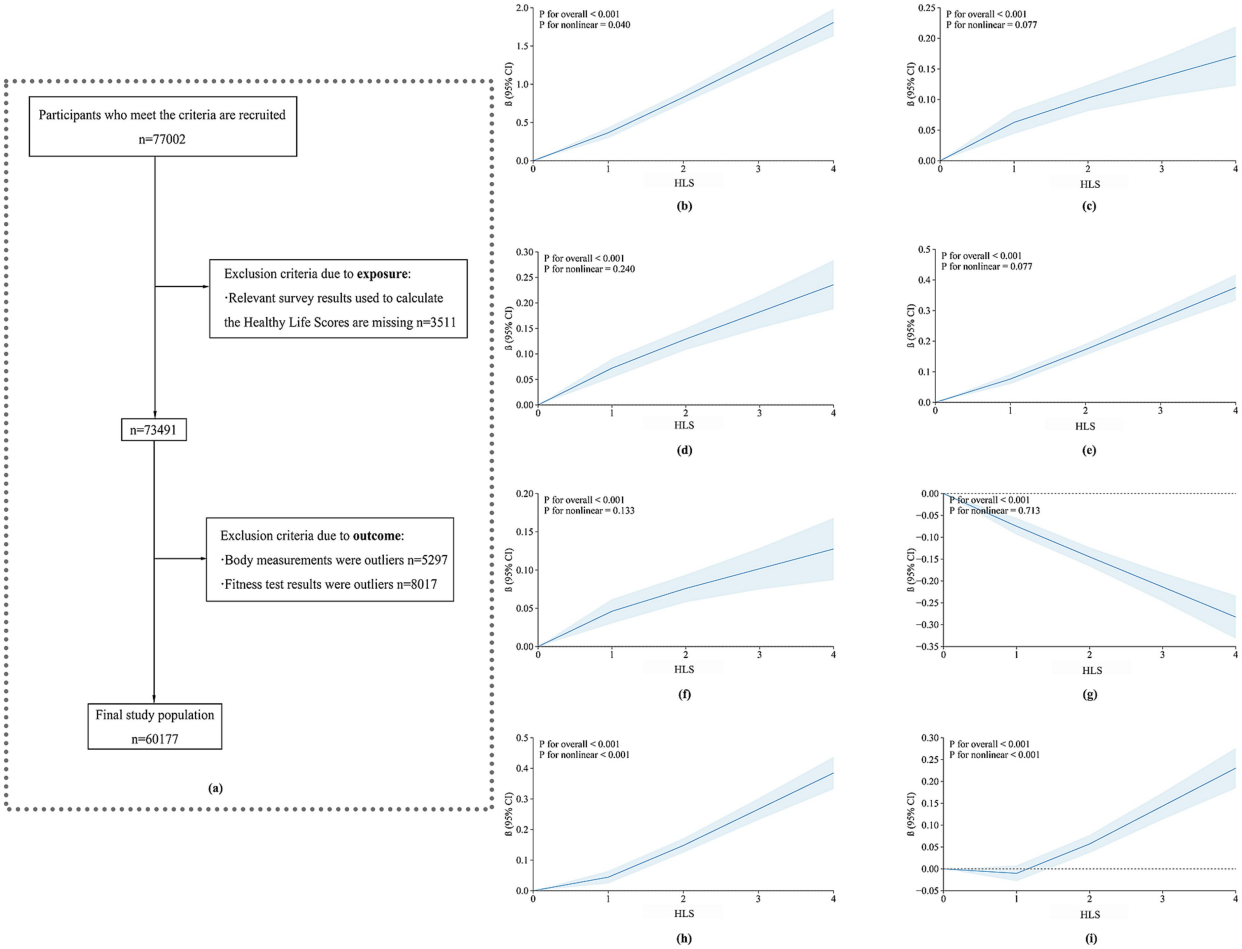
**(a)** Flow chart for recruiting participants. **(b–i)** Association between HLS and the PFI or individual physical fitness tests using a restricted cubic spline regression model: **(b)** PFI; **(c)** grip strength; **(d)** standing long jump; **(e)** 30-s sit-ups; **(f)** sit-and-reach; **(g)** 50 m dash; **(h)** 20 m SRT z-score; **(i)** 20-s repeated straddling. *β*-coefficient (95% CI) was adjusted for age, sex (girl or boy), residence (27 different provinces), parental occupation (Civil servant; Worker; Company staff; Go into business; Peasant; Other), parental education (less than primary high school; high school or equivalent; some college or above), household income (less than RMB 2,000; RMB 2,001 to 5,000; RMB 5001–8,000; more than RMB 8,000), BMI. The data were fitted using a linear regression model with 3 knots at the 10th, 50th, 90th percentiles of HLS (reference: 10th percentile). Solid lines represent *β*-coefficient, while shaded areas indicate 95% CIs. CIs, confidence intervals.

Of the initial 77,022 participants, 3,511 were excluded due to missing data on key lifestyle factors, including diet, physical activity, screen time, and sleep. To maintain the integrity and scientific validity of our physical fitness test data, an additional 5,297 participants were excluded for unreasonable anthropometric values and 8,017 for extreme fitness test results, defined as values below the 5th or above the 95th percentile for age and gender. The final analysis comprised 60,177 participants (47.8% boys), as detailed in the participant flowchart (Figure 1a).

### 2.3 HLS (exposures)

Data on participants’ living conditions were collected via structured interviews with guardians. The HLS was developed based on the published scoring models (14) with modification for our study. The score evaluates four key lifestyle dimensions based on existing recommendation guidelines or standards: Healthy diet, physical activity, screen time, and sleep patterns (Supplementary Table S1). Each behavior was scored as 1 for a healthy practice, or 0 for a poor practice. The definitions of healthy behaviors were as follows: (1) Healthy Eating: defined as consuming three or more food groups daily and limiting sugar-sweetened beverages to no more than once per week; (2) Sleep Duration: ≥8 h per day was regarded as adequate, corresponding to the lower bound of the recommended sleep duration for the predominant age groups in the study population; (3) Screen Time: keeping daily screen exposure within age-appropriate limits; (4) Physical Activity: Participating in physical activities at least three times per week with each session lasting over 1 h. It is also important to note that the measure of physical activity frequency relied on parent-reported engagement in organized exercise. Physical activity in young children is often unstructured and intermittent in nature, which may not be fully captured by this metric. HLS is the sum of scores of four key lifestyle dimensions, ranged from 0 to 4 points, where a higher score indicates a healthier lifestyle.

### 2.4 Physical fitness assessment and index (outcomes)

Physical fitness tests followed national standards, with 1–2 sports science professionals and 4–5 certified physical education instructors per setting for consistent measurements. All equipment was calibrated before testing, and assessments were conducted at similar times of day. The tests included grip strength, standing long jump, 30-s sit-ups, sit-and-reach, 50 m dash, 20-s repeated straddling, and the 20 m shuttle run test (20 m SRT). To allow comparisons across age and gender, Z-scores for each test were calculated using the formula. The primary outcome, the Physical Fitness Index (PFI) (15), was derived by summing the Z-scores of six tests and subtracting the Z-score of the 50 m dash, since a shorter time indicates better performance. Additionally, individual test Z-scores were analyzed as secondary outcomes since each test targets specific fitness areas. Grip strength reflects upper limb muscle strength (16), and is an indicator of nutritional status and cardio-metabolic risk (17), Standing long jump assesses lower body strength and core activation (18). The 30-s sit-ups measures muscular endurance. Sit-and-reach test evaluates muscular flexibility. The 50 m dash reflects speed and power (19). The 20-s repeated straddling evaluates agility (20). The 20 m SRT serves as a marker for cardiorespiratory fitness and health (21).

### 2.5 Statistical analysis

Multivariate linear regression models were used to examine the association between HLS and physical fitness outcomes. The primary outcome was the overall PFI, while the individual physical fitness Z-scores were treated as exploratory outcomes. All models were adjusted for potential confounders, including age, gender, region, parental occupation, parental education, household income, and BMI. Regression coefficients (*β*) and 95% confidence intervals (CIs) were reported. To account for multiple comparisons among outcomes, the Benjamini-Hochberg false discovery rate (FDR) correction was applied, and FDR-adjusted *p*-values are reported alongside raw p-values.

Subgroup analyses evaluated the associations of each lifestyle factors and physical fitness outcome. Restricted Cubic Spline (RCS) models were used to explore potential nonlinear associations between HLS and physical fitness measures. Comparisons across different numbers of healthy lifestyle factors were made using individuals with zero qualifying factors as the reference group. Geographic variations in the relationship between HLS and PFI across provinces were visualized using heatmaps, based on regression coefficients and associated health scores.

We conducted several sensitivity analyses to confirm our results’ reliability: First, we used an alternative score, Health Lifestyle Score-D (HLS-D), incorporating dietary diversity, sleep duration, screen time and physical activity, in regression and RCS analysis (Supplementary Table S1). Second, multiple imputation was employed to address missing data, and the models were refitted using the imputed dataset. Multiple imputation was performed via the mice package in R, with the random seed set to 123 to ensure reproducibility. The imputation model included all variables in the primary analysis, namely HLS, exercise duration, sleep duration, screen time, healthy eating score, PFI, the seven physical fitness test scores, age, sex, residence, parental occupation, parental education, monthly household income, and BMI. Missing data were assumed to be missing at random (MAR). A total of 20 imputed datasets were generated, and the estimates from each dataset were combined according to Rubin’s rules. Third, the analyses were repeated after excluding obese participants to evaluate extreme BMI values effects. Fourth, linear models were performed after excluding BMI as a covariate. Fifth, given that the age range of 7–18 years encompasses distinct developmental stages, we performed age-stratified subgroup analyses, by categorizing participants as children (7–12 years) and adolescents (13–18 years), to explore whether the associations varied across developmental periods. All analyses were performed using IBM SPSS Statistics 23.0 and R 4.4.1, with significance at *p*-value < 0.05.

## 3 Results

### 3.1 Characteristics of participants

A total of 60,177 children and adolescents (47.77% boys and 52.23% girls) with a mean age of 14.0 (5.0) years were included (Table 1). Among participants, 20.49% of fathers and 17.45% of mothers obtained a bachelor’s degree or higher. The mean PFI score was 0.08 (4.90) points. Average z-scores (SD) were: grip strength, −0.02 (1.27); 30-s sit-ups, −0.09 (1.23); standing long jump, 0.00 (1.27); sit-and-reach, −0.03 (1.17); 50 m dash, −0.11 (1.22); 20 m SRT, −0.09 (1.41); and 20-s repeated straddling, 0.09 (1.20). For the HLS, 22.07% scored 0, 42.97% scored 1, 26.83% scored 2, 6.88% scored 3, and 1.24% scored 4.

**Table 1:** Characteristics of participants.

Characteristics	Participants (*n* = 60,177)
Age	14.0 (5.0)^a^
Sex assigned at birth	
Boys	28,746 (47.77) ^b^
Girls	31,431 (52.23) ^b^
Mother’s education level	
Secondary high school or lower	31,379 (52.14) ^b^
High school	18,295 (30.40) ^b^
Bachelor’s or higher	10,503 (17.45) ^b^
Mother’s employment	
Civil servant	6,364 (10.58) ^b^
Worker	8,622 (14.33) ^b^
Company staff	12,450 (20.69) ^b^
Business owner (including self-employed)	7,507 (12.47) ^b^
Farmer	10,193 (16.94) ^b^
Other	15,041(24.99) ^b^
Father’s education level	
Secondary high school or lower	28,292 (47.01) ^b^
High school	19,556 (32.50) ^b^
Bachelor’s or higher	12,329 (20.49) ^b^
Father’s employment	
Civil servant	6,665 (11.08) ^b^
Worker	12,339 (20.50) ^b^
Company staff	12,141 (20.18) ^b^
Business owner (including self-employed)	9,030 (15.01) ^b^
Farmer	9,065 (15.06) ^b^
Other	10,937 (18.17) ^b^
Household income, RMB/month	
≤2000	7,576 (12.59) ^b^
2001–5,000	22,196 (36.88) ^b^
5,001–8,000	17,211 (28.60) ^b^
>8,000	13,194 (21.93) ^b^
Physical fitness status	
Body muscle strength	
Grip strength z-score	−0.02 (1.27)^a^
Standing long jump z-score	0.00 (1.27)^a^
30-s sit-ups z-score	−0.09 (1.23)^a^
Sit-and-reach z-score	−0.03 (1.17)^a^
50 m dash z-score	−0.11 (1.22)^a^
20 m SRT z-score	−0.09 (1.41)^a^
20-s repeated straddling z-score	0.09 (1.20)^a^
PFI	0.08 (4.90)^a^
Healthy lifestyles	
Healthy eating	
0	53,290 (88.56)^b^
1	6,887 (11.44)^b^
Sleep duration	
0	45,171 (75.06)^b^
1	15,006 (24.94)^b^
Screen time	
0	54,488 (90.55)^b^
1	5,689 (9.45)^b^
Physical activity	
0	27,689 (46.01)^b^
1	32,488 (53.99)^b^
HLS	
0	13,284 (22.07)^b^
1	25,861 (42.97)^b^
2	16,146 (26.83)^b^
3	4,142 (6.88)^b^
4	744 (1.24)^b^

^a^Data shown as median (IQR). ^b^ Data shown as relative frequency (%).

### 3.2 Association between HLS and physical fitness

Figures 1b–i illustrates the RCS models, adjusted for potential confounders, demonstrating significant associations between HLS and the overall PFI and individual physical fitness tests (all P for overall < 0.001). Nonlinear relationships were observed for PFI and the 20 m SRT (all P for nonlinear < 0.05), with a distinct inflection point at an HLS of 1, after which the slope increased notably. Similarly, the association between HLS and the 20-s repeated straddling followed a J-shaped curve (P for nonlinear < 0.001), with an HLS inflection point at 1. In contrast, linear relationships were found for PFI and grip strength, standing long jump, 30-s sit-ups, sit-and-reach and the 50 m dash (all P for nonlinear > 0.05).

Each one-point increase in HLS was significantly linked to a 0.43-point increase in PFI (95% CI: 0.39 to 0.47, *p* < 0.001, Table 2). Significant positive associations were also observed for grip strength (0.05, 95% CI: 0.04 to 0.06), 30-s sit-ups (0.09, 95% CI: 0.08 to 0.10), standing long jump (0.06, 95% CI: 0.05 to 0.07), sit-and-reach (0.04, 95% CI: 0.03 to 0.05), 20 m SRT (0.08, 95% CI: 0.07 to 0.09) and 20-s repeated straddling (0.04, 95% CI: 0.03 to 0.05, all *p* < 0.001). Conversely, a lower HLS was associated with a higher Z-score for the 50 m dash (−0.07, 95% CI: −0.08 to −0.06, p < 0.001). All variance inflation factor (VIF) values were below 5, indicating no serious multicollinearity (Supplementary Table S2).

**Table 2:** The linear regression model of healthy lifestyle score and PFI and individual physical fitness domains.

Variables	Crude model				Multivariable-adjusted model^a^			
	*β*-coefficient	95% CI	*p*-value	Adjusted *p*-value (FDR)^b^	*β*-coefficient	95% CI	*p*-value	Adjusted *p*-value (FDR)^b^
PFI	0.47	(0.43, 0.50)	<0.001*	<0.001*	0.43	(0.39, 0.47)	<0.001*	<0.001*
Grip strength	0.03	(0.02, 0.04)	<0.001*	<0.001*	0.05	(0.04, 0.06)	<0.001*	<0.001*
30-s sit-ups	0.09	(0.09, 0.10)	<0.001*	<0.001*	0.09	(0.08, 0.10)	<0.001*	<0.001*
Standing long jump	0.05	(0.04, 0.06)	<0.001*	<0.001*	0.06	(0.05, 0.07)	<0.001*	<0.001*
Sit-and-reach	0.04	(0.03, 0.05)	<0.001*	<0.001*	0.04	(0.03, 0.05)	<0.001*	<0.001*
50 m dash	−0.06	(−0.06, −0.05)	<0.001*	<0.001*	−0.07	(−0.08, −0.06)	<0.001*	<0.001*
20 m SRT	0.07	(0.06, 0.08)	<0.001*	<0.001*	0.08	(0.07, 0.09)	<0.001*	<0.001*
20-s repeated straddling	0.03	(0.02, 0.04)	<0.001*	<0.001*	0.04	(0.03, 0.05)	<0.001*	<0.001*

CI, confidence interval; PFI, Physical fitness index. ^a^Adjusted for age, sex (girl or boy), residence (27 different provinces), parental occupation (Civil servant; Worker; Company staff; Business owner (including self-employed); Farmer; Other), parental education (less than primary high school; high school or equivalent; some college or above), household income (less than RMB 2,000; RMB 2,001 to 5,000; RMB 5001–8,000; more than RMB 8,000), BMI. ^b^*p*-values were adjusted for multiple comparisons using the Benjamini–Hochberg false discovery rate (FDR) procedure. ^*^The results were statistically different, *p* < 0.05.

### 3.3 Relationship between individual lifestyles and physical fitness

**Figure 2:**
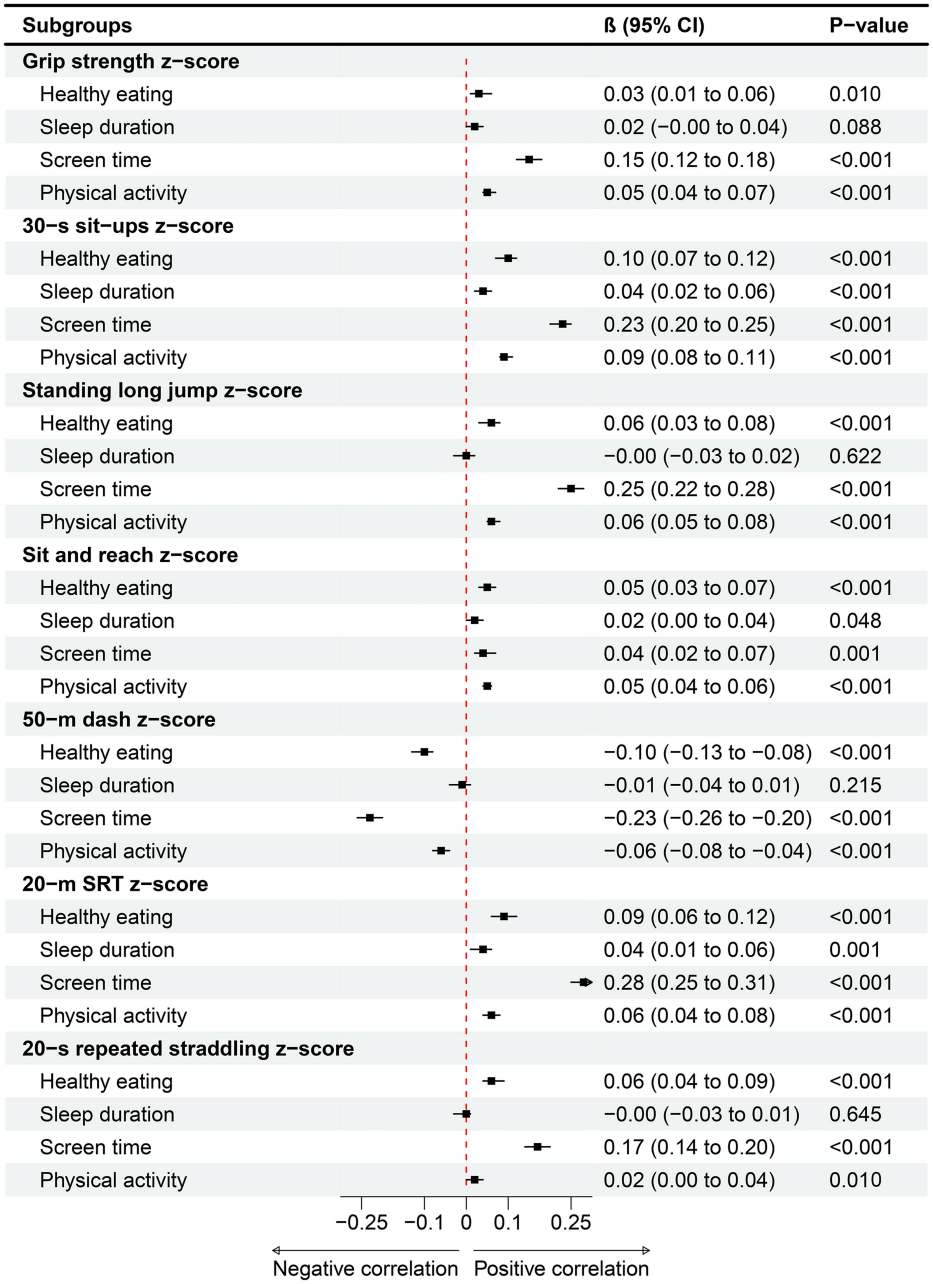
The association of five individual healthy lifestyles with physical fitness domains among children and adolescents. The model was adjusted for age, sex, residence, parental occupation, parental education, monthly household income, BMI.

As shown in Figure 2, after adjusting for confounders, healthy eating was positively associated with grip strength (0.03, 95% CI: 0.01 to 0.06), 30-s sit-ups (0.10, 95% CI: 0.07 to 0.12), standing long jump (0.06, 95% CI: 0.03 to 0.08), sit-and-reach (0.05, 95% CI: 0.03 to 0.07), 20 m SRT (0.10, 95% CI: 0.07 to 0.12), and 20-s repeated straddling (0.06, 95% CI: 0.04 to 0.08) (all *p* < 0.01). Lower screen time were linked to better performance in grip strength (0.15, 95% CI: 0.12 to 0.18), 30-s sit-ups (0.23, 95% CI: 0.20 to 0.25), standing long jump (0.25, 95% CI: 0.22 to 0.28), sit-and-reach (0.04, 95% CI: 0.02 to 0.07), 20 m SRT(0.28, 95% CI: 0.25 to 0.31), and 20-s repeated straddling (0.17, 95% CI: 0.14 to 0.20) (all *p* < 0.001). Additionally, physical activity were positively associated with higher z-score in grip strength (0.05, 95% CI: 0.04 to 0.07), 30-s sit-ups (0.09, 95% CI: 0.08 to 0.11), standing long jump (*β* = 0.06, 95% CI: 0.05 to 0.08), sit-and-reach (0.05, 95% CI: 0.04 to 0.06), 20 m SRT(0.06, 95% CI: 0.04 to 0.08), and 20-s repeated straddling (0.02, 95% CI: 0.00 to 0.04) (all *p* < 0.01). Furthermore, longer sleep duration was positively associated with 30-s sit-ups (0.04, 95% CI: 0.02 to 0.06), sit-and-reach (0.02, 95% CI: 0.00 to 0.04), and 20 m SRT (0.04, 95% CI: 0.01 to 0.06) (all *p* < 0.05). Conversely, healthy eating, lower screen time, and higher physical activity were all associated with faster 50 m dash times (−0.10, 95% CI: −0.12 to −0.08; −0.10, 95% CI: −0.13 to −0.08; −0.23, 95% CI: −0.26 to −0.20; −0.06, 95% CI: −0.08 to −0.04, all *p* < 0.001).

**Figure 3:**
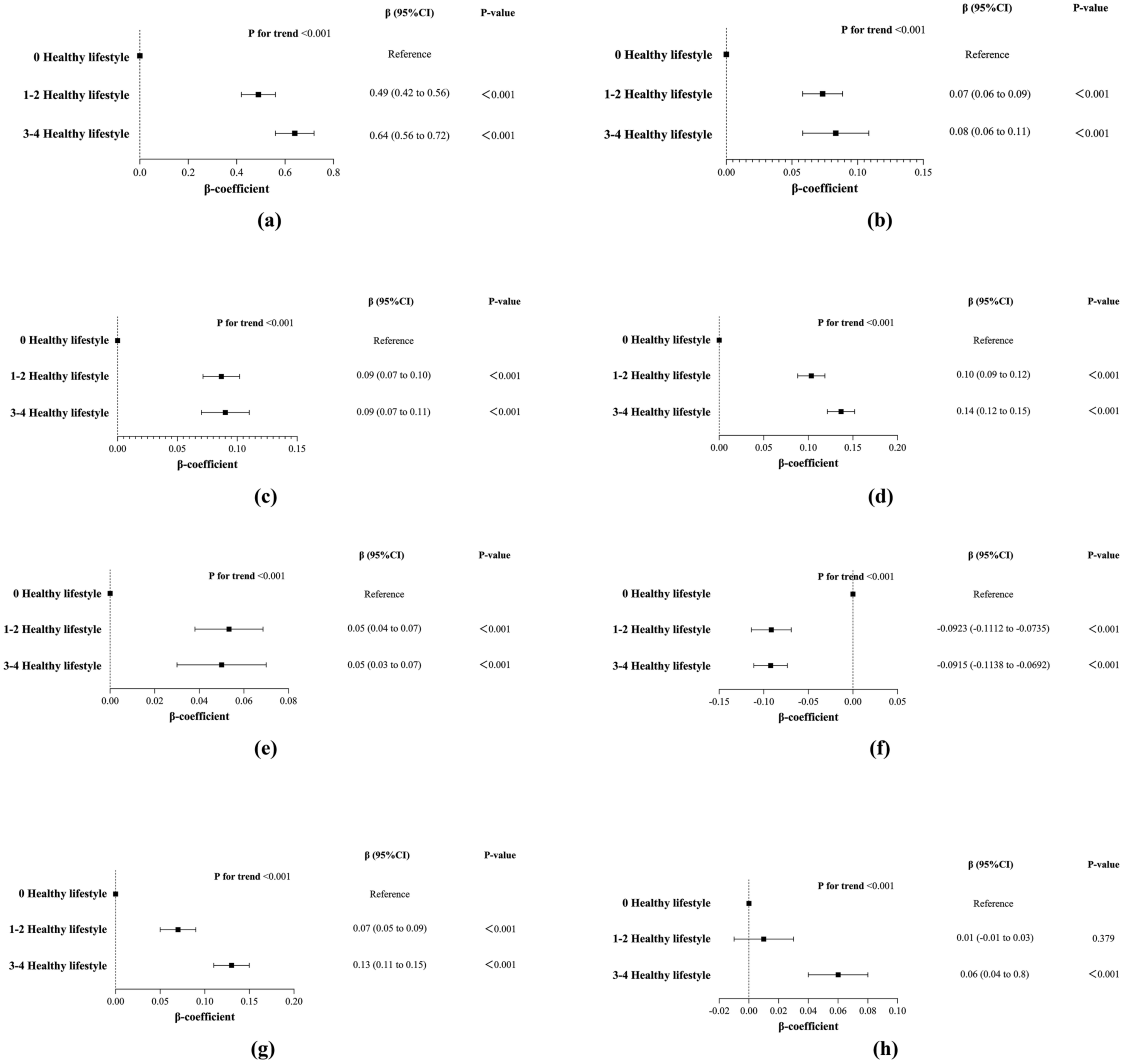
The associations between the numbers of healthy lifestyles and physical fitness among children and adolescents. **(a)** PFI; **(b)** Grip strength; **(c)** Standing long jump; **(d)** 30-s sit-ups; **(e)** Sit-and-reach; **(f)** 50 m dash; **(g)** 20 m SRT; **(h)**20-s repeated straddling. Calculations are performed using a multiple linear regression model.

Children with 3–4 healthy lifestyle factors exhibited greater improvements in PFI, grip strength, standing long jump, 30-s sit-ups, and 20 m SRT compared to those with 1–2 healthy lifestyle factors (Figures 3a–d,g). Conversely, sit and reach and 50 m dash were better in children with 1–2 healthy lifestyle factors than those with 3–4 factors (Figures 3e,f). No significant difference was observed in 20-s repeated straddling performance between children with no factors and those with 1–2 (*p* = 0.379, see Figure 3h). Furthermore, performance improved in all fitness tests as the number of healthy lifestyle factors increases (all P for trend < 0.001).

### 3.4 Regional distribution of HLS and PFI

**Figure 4:**
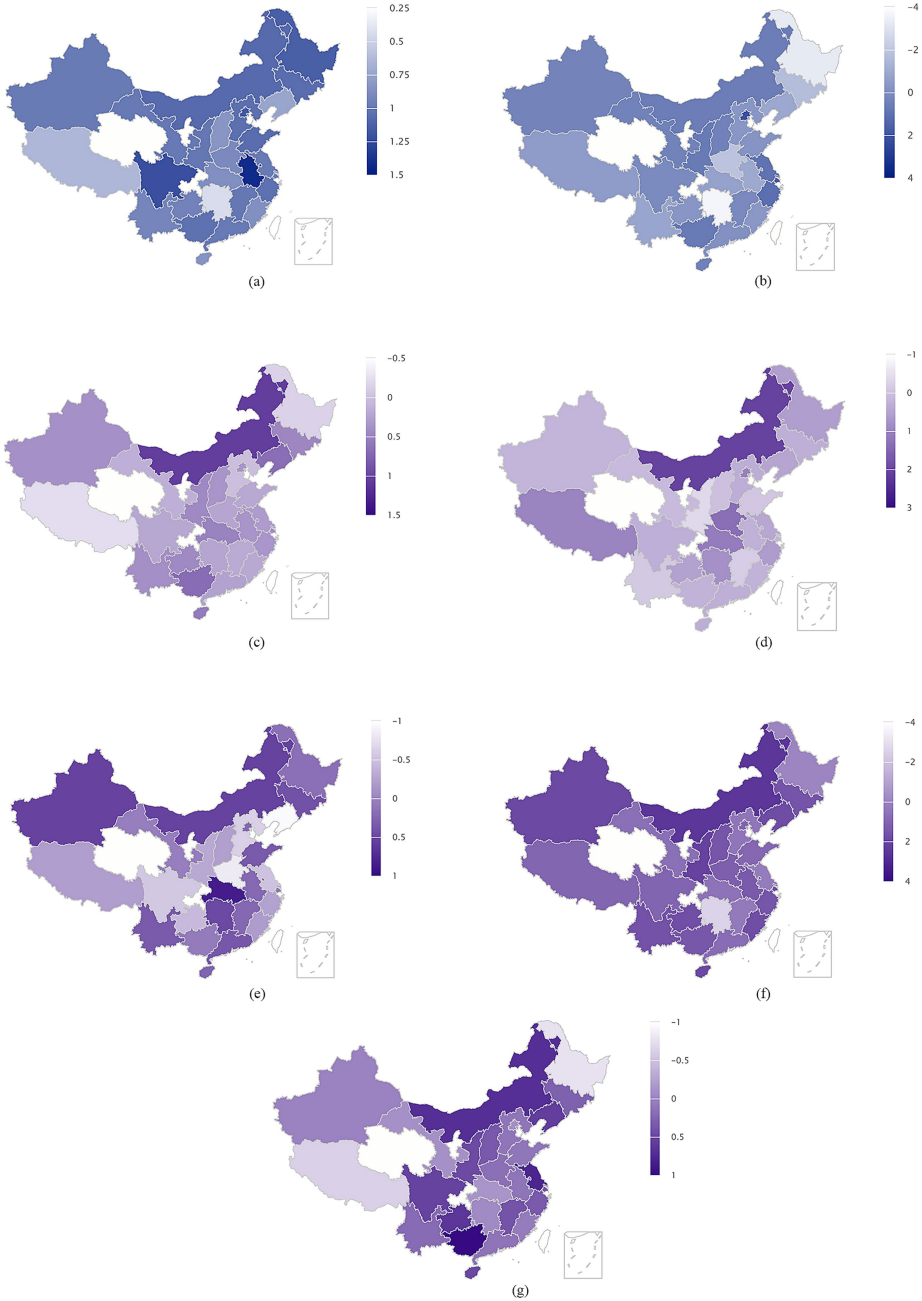
Heat maps depicting regional variations in healthy lifestyle behaviors scores and PFI across 27 provinces of China. **(a)** HLS; **(b)** PFI; **(c)** *β* values for HLS and PFI; **(d)** *β* values for Health eating and PFI; **(e)** *β* values for Sleep duration and PFI; **(f)** *β* values for Physical activity and PFI; **(g)** *β* values for Screen time and PFI. The darker the color, the larger the value; white areas represent regions of missing data. China’s 31 provinces were categorized into Eastern, Central, and Western regions for analysis, following the standard socioeconomic and geographical classification used by the National Bureau of Statistics of China.

Across China, Anhui (1.42) and Sichuan (1.22) ranked highest in HLS (Figure 4a). For PFI, Beijing (PFI = 2.19) and Shanghai (PFI = 1.78) led the rankings (Figure 4b). Notably, inner Mongolia exhibited the strongest association between HLS (*β* = 1.07), healthy eating (*β* = 2.06) and PFI (Figures 4c,d). The associations between individual lifestyle behaviors and PFI demonstrated notable regional variation across China (Figures 4e–g).

### 3.5 Sensitivity analyses

Substituting HLS-D yielded significant positive correlations with most fitness tests and PFI, and a negative correlation with the 50 m dash with nonlinear associations observed. After multiple imputations, the associations remained persistent between HLS and individual tests and PFI (Supplementary Figure S1b). In stratified analyses, higher HLS remained significantly associated with improved fitness outcomes among non-obese youth. Age-stratified subgroup analyses demonstrated that the associations between HLS and PFI, as well as six of the seven individual fitness tests, remained significant among children (7–12 years), with the exception of handgrip strength. Among adolescents (13–18 years), HLS was significantly associated with PFI and all seven fitness tests outcomes. To address the potential over-adjustment bias from BMI acting as a mediator, we performed a sensitivity analysis without adjustment for BMI (Supplementary Table S3). The associations between HLS and PFI, as well as individual physical fitness test scores, remain consistent. Additionally, participants with 3–4 healthy lifestyle factors exhibited greater improvements in PFI than those with 1–2 factors (Supplementary Figure S1). Similar dose–response patterns were observed in both children and adolescents (Supplementary Figure S2).

## 4 Discussion

In this nationally representative study of Chinese children and adolescents, higher HLS scores were consistently associated with enhanced PFI and performance across multiple fitness domains. Notably, a graded relationship was observed, where the number of healthy lifestyle factors corresponded with improved fitness test outcomes. Among the individual lifestyle behaviors assessed, limited screen time exhibited the strongest association with higher PFI and better performance in specific fitness tests, followed by healthy eating, physical activity, and adequate sleep. These findings highlight the necessity of a comprehensive approach to lifestyle interventions for optimizing physical fitness in youth.

To the best of our knowledge, this is the first nationally representative investigation using multidimensional fitness assessments rather than self-reported data or isolated fitness measures, to explore the relationship between lifestyle behaviors and physical fitness. For example, one cross-sectional study of children aged 7–17 year identified that the combination of adequate sleep, less screen time and sufficient moderate-to-vigorous physical activity was linked to self-reported general physical fitness, CRF, muscular strength, speed and agility, as well as flexibility (22). Similarly, a cohort study in adolescents demonstrated that higher healthy lifestyle scores, incorporating physical activity, reduced sedentary time, healthy diet, and sufficient sleep, were associated with improved CRF over a 24-month period (11).

Our study identified a significant trend in which the PFI and individual fitness domains improved as the number of healthy lifestyle factors increased. Similarly, a prospective cohort study demonstrated that adherence to a greater number of healthy lifestyle factors, including diet, smoking, physical activity, alcohol consumption, and BMI, was associated with lower risks of all-cause, cancer, and cardiovascular mortality (23). Furthermore, systematic reviews summarizing large-scale cohort studies, have consistently shown an inverse relationship between the number of healthy lifestyle factors and all-cause mortality risk, with a combination of at least four healthy behaviors linked to a 66% reduction in the risk of all-cause mortality (24). Our results extend these findings by demonstrating that a comprehensive healthy lifestyle can have a synergistic effect on enhancing physical fitness across multiple domains in children and adolescents.

Prior research among 8,136 Chinese adolescents has demonstrated significant associations between prolonged screen time and poorer general physical fitness (13). Similarly, a two-year longitudinal study reported that excessive mobile phone use (>2 h/day) in children was linked to reduced CRF (25). In our study, limited screen time, defined as <1 h/day for those under 18 and <2 h/day for those aged 18 and older, has been most strongly associated with higher PFI and improved performance in individual fitness tests. Although only 9.45% of participants met these strict screen time criteria, sensitive analyses confirmed that the association remained robust when a criterion of < 2 h/day was applied to youth (Supplementary Figure S1). The proportion of 23.85% comparable to the previously reported 25.3% of Chinese children and adolescents (26). Furthermore, a national survey indicated a downward trend in compliance with the < 2 h of screen time among Chinese children and adolescents from 2017 to 2019 (27), highlighting the growing public health challenge of managing screen exposure among youth.

Previous studies reinforce that healthy dietary habits are closely linked to improved physical fitness. For example, adherence to the Mediterranean diet has been associated with higher fitness levels and more favorable body composition in adolescents (28). Similarly, findings from the Framingham Heart Study revealed that better dietary quality correlates with improved CRF in adults (29). Additional cross-sectional study found that higher consumption of eggs and dairy products, coupled with lower sugar-sweetened beverages intake, is associated with superior physical fitness, particularly in strength and endurance (30). In addition, previous research has revealed that 22.61% of children aged 3–17 years consumed fewer than eight food groups over 3 days (31). In our study, the healthy diet prevalence was lower at 11.44%, based on our criteria integrating dietary diverse and sugary drink limitation. These findings provide insights into targeted interventions that promote varied nutrition intake while reducing sugary sweetened beverages consumption, which may, in turn, yield benefits for physical fitness in pediatric populations.

Evidence supports benefits of physical activity on physical fitness across various populations, including adolescents (32), older adults (33) and sedentary individuals (34). In our study, approximately half of youth did not meet the recommended intensity and duration for physical activity, which aligns with a cross-sectional study reporting that 46.7% of participants did not engage in physical activity (35). These observations underscore persistent challenge of promoting adequate physical activity among youth, and the need for strategies to foster healthier behaviors.

Our study reveals significant regional variations in the associations between healthy lifestyles and physical fitness. Inner Mongolia exhibited the strongest relationship between HLS and PFI, suggesting that targeted lifestyle interventions in specific regions could yield benefits. These differences likely reflect unique cultural, environmental, or socioeconomic factors, highlighting the need for further investigation into these mechanisms to develop tailored intervention strategies.

Strengths of our study include a nationally representative, large-sample design, which enhances robustness and generalizability. Moreover, our multidimensional approach provides novel insights into how lifestyle factors collectively influence physical fitness, surpassing previous research that often relied on self-reported outcomes or single-factor analyses. However, this study has several limitations. First, the self-reported nature of lifestyle behaviors in this study is subject to measurement error. However, we applied a validated questionnaire to improve estimates of usual lifestyle behaviors. Second, the exclusion of participants with incomplete data may have introduced selection bias, but we conducted multiple sensitive analyses to confirm the robustness of our results. Third, the cross-sectional study design limits causal inference, highlighting the need for longitudinal research to better establish relationships. Fourth, the data used in this study were collected in 2015–2016, which may limit the direct generalizability of the findings to the present day. Although lifestyle patterns and social environments may have changed over time, the healthy lifestyle behaviors examined in this study remain central components of current public health recommendations, and their associations with physical fitness are expected to remain broadly relevant. Finally, potential residual confounding cannot be entirely ruled out.

## 5 Conclusion

This nationally representative cross-sectional study implicates the critical role of adopting healthy lifestyle behaviors in enhancing the physical fitness of children and adolescents. Our findings indicate that simultaneous improvements across multiple lifestyle factors, can yield synergistic benefits that exceed the effects of addressing individual behaviors in isolation. These results provide a compelling rationale for the developing and implementing evidence-based guidelines and interventions to foster healthy lifestyle habits, ultimately promote the well-being of young populations. Future research should use recent and nationally representative data to validate these findings in contemporary populations. In addition, longitudinal and intervention studies are warranted to further clarify causal relationships and to support the development of targeted strategies for improving lifestyle behaviors.

## Data Availability

The datasets used and/or analyzed during the current study are available from the corresponding author on reasonable request.
