# Supplementary material for: Association of healthy lifestyle and physical fitness in children: a nationwide population-based cross-sectional study
Source: Front Public Health. 2026 Jul 8;14:1885065. doi: 10.3389/fpubh.2026.1885065 (PMC13388834; doi:10.3389/fpubh.2026.1885065)
Supplement: Supplementary file 1 [file Table_1.DOCX]

Supplementary Material

# Supplementary Data-STROBE Statement

## STROBE Statement—Checklist of items that should be included in reports of cross-sectional studies

|  | **Item No** | **Recommendation** | **Location where item is reported** |
| --- | --- | --- | --- |
| **Title and abstract** | 1 | (*a*) Indicate the study’s design with a commonly used term in the title or the abstract | Page 1 |
|  |  | (*b*) Provide in the abstract an informative and balanced summary of what was done and what was found | Page 3 |
| **Introduction** | | | |
| Background/rationale | 2 | Explain the scientific background and rationale for the investigation being reported | Page 5–6 |
| Objectives | 3 | State specific objectives, including any prespecified hypotheses | Page 6 |
| **Methods** | | | |
| Study design | 4 | Present key elements of study design early in the paper | Page 7 |
| Setting | 5 | Describe the setting, locations, and relevant dates, including periods of recruitment, exposure, follow-up, and data collection | Page 7–8 |
| Participants | 6 | (*a*) Give the eligibility criteria, and the sources and methods of selection of participants | Page 7–8 |
| Variables | 7 | Clearly define all outcomes, exposures, predictors, potential confounders, and effect modifiers. Give diagnostic criteria, if applicable | Page 8–10 |
| Data sources/ measurement | 8* | For each variable of interest, give sources of data and details of methods of assessment (measurement). Describe comparability of assessment methods if there is more than one group | Page 8–10 |
| Bias | 9 | Describe any efforts to address potential sources of bias | Page 7–8, 11 |
| Study size | 10 | Explain how the study size was arrived at | Page 7–8 |
| Quantitative variables | 11 | Explain how quantitative variables were handled in the analyses. If applicable, describe which groupings were chosen and why | Page 10–11 |
| Statistical methods | 12 | (*a*) Describe all statistical methods, including those used to control for confounding | Page 10–11 |
|  |  | (*b*) Describe any methods used to examine subgroups and interactions | Page 10–11 |
|  |  | (*c*) Explain how missing data were addressed | Page 8, 10–11 |
|  |  | (*d*) If applicable, describe analytical methods taking account of sampling strategy | Page 7–8, 10–11 |
|  |  | (*e*) Describe any sensitivity analyses | Page 11 |
| **Results** | | | |
| Participants | 13* | (a) Report numbers of individuals at each stage of study—eg numbers potentially eligible, examined for eligibility, confirmed eligible, included in the study, completing follow-up, and analysed | Page 8 |
|  |  | (b) Give reasons for non-participation at each stage | Page 8 |
|  |  | (c) Consider use of a flow diagram | Page 8 |
| Descriptive data | 14* | (a) Give characteristics of study participants (eg demographic, clinical, social) and information on exposures and potential confounders | Page 11 |
|  |  | (b) Indicate number of participants with missing data for each variable of interest | Supplementary material: Table S4 |
| Outcome data | 15* | Report numbers of outcome events or summary measures | Table 1 |
| Main results | 16 | (*a*) Give unadjusted estimates and, if applicable, confounder-adjusted estimates and their precision (eg, 95% confidence interval). Make clear which confounders were adjusted for and why they were included | Page 12, Table 2 |
|  |  | (*b*) Report category boundaries when continuous variables were categorized | Page 11–12 |
|  |  | (*c*) If relevant, consider translating estimates of relative risk into absolute risk for a meaningful time period | Not applicable |
| Other analyses | 17 | Report other analyses done—eg analyses of subgroups and interactions, and sensitivity analyses | Page 12–15 |
| **Discussion** | | | |
| Key results | 18 | Summarise key results with reference to study objectives | Page 15 |
| Limitations | 19 | Discuss limitations of the study, taking into account sources of potential bias or imprecision. Discuss both direction and magnitude of any potential bias | Page 18–19 |
| Interpretation | 20 | Give a cautious overall interpretation of results considering objectives, limitations, multiplicity of analyses, results from similar studies, and other relevant evidence | Page 15–18 |
| Generalisability | 21 | Discuss the generalisability (external validity) of the study results | Page 15–18 |
| **Other information** | | | |
| Funding | 22 | Give the source of funding and the role of the funders for the present study and, if applicable, for the original study on which the present article is based | Page 24 |

*Give information separately for exposed and unexposed groups.

**Note:** An Explanation and Elaboration article discusses each checklist item and gives methodological background and published examples of transparent reporting. The STROBE checklist is best used in conjunction with this article (freely available on the Web sites of PLoS Medicine at http://www.plosmedicine.org/, Annals of Internal Medicine at http://www.annals.org/, and Epidemiology at http://www.epidem.com/). Information on the STROBE Initiative is available at www.strobe-statement.org.

# Supplementary Figures and Tables


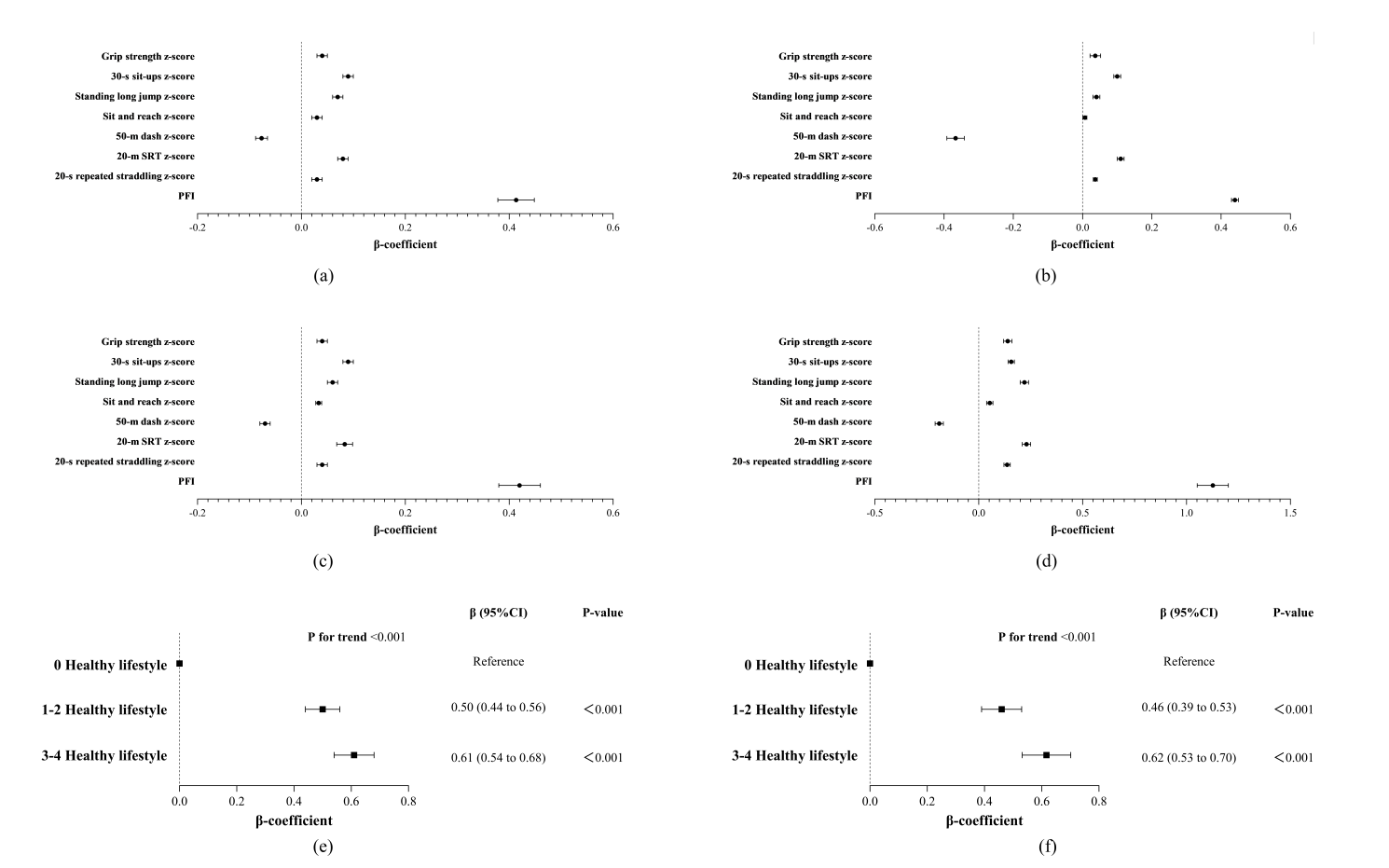


**Fig. S1** The results of sensitivity analyses. (a) The linear regression model of HLS-D with PFI and individual physical test; (b) he linear regression model of HLS with PFI and individual physical tests among children and adolescents with imputated data; (c) The linear regression model of HLS with PFI and individual physical tests among children and adolescents without obesity; (d) The linear regression model of healthy lifestyle score and PFI and individual physical test based on Screen Time-S; (e) The association between the number of healthy lifestyle behaviors and the PFI among children and adolescents with imputed data; (f) The association between the number of healthy lifestyle and the PFI among children without obesity. The linear regression model was adjusted for age, sex, residence, parental occupation, parental education, monthly household income, BMI.


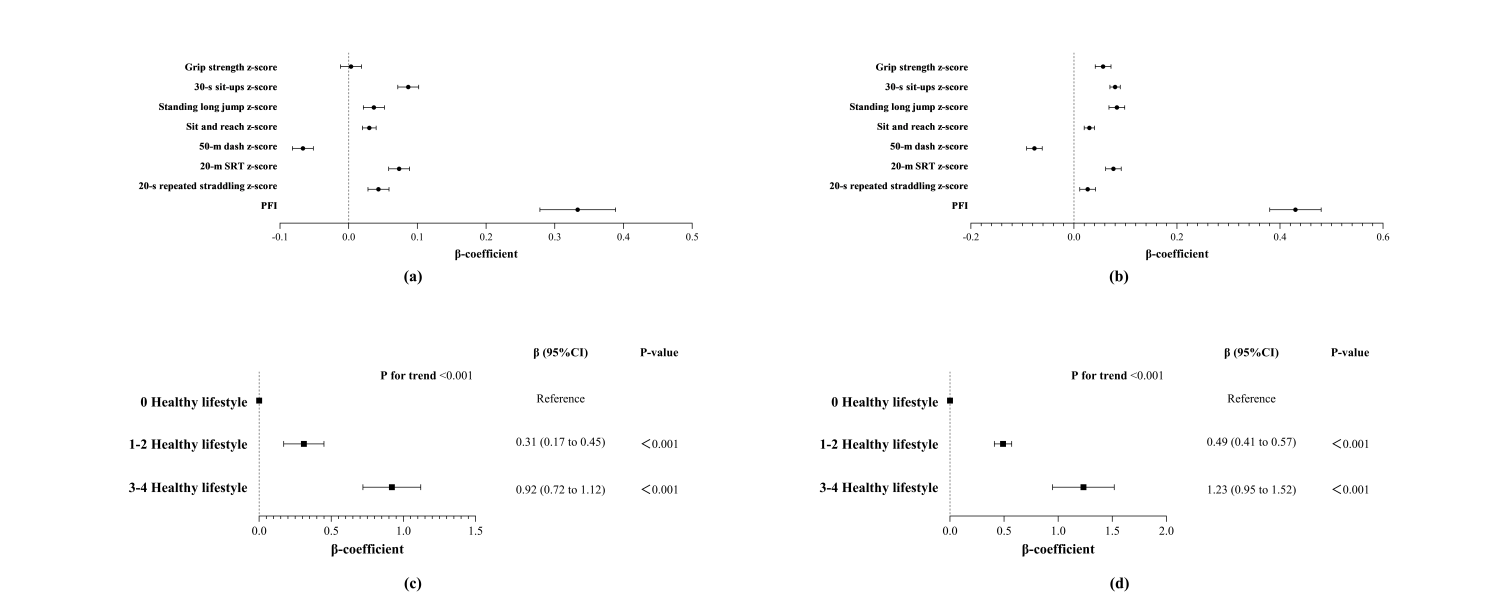


**Fig. S2** Results of the age‑stratified analyses. (a) Linear regression models of the association between HLS and PFI and individual physical fitness test measures in children (7–12 years). (b) Linear regression models of the association between HLS and PFI and individual physical fitness test measures in adolescents (13–18 years). (c) Association between the number of healthy lifestyle behaviors and PFI in children. (d) Association between the number of healthy lifestyle behaviors and PFI in adolescents. The linear regression model was adjusted for age, sex, residence, parental occupation, parental education, monthly household income, BMI.

**Table S1.** The calculation criteria for Healthy Lifestyle Score

| **Subject** | **scoring criteria** | **Point** |
| --- | --- | --- |
| Healthy Eating | < 3 food groups^*^/day or sugary drink > 1 time/week | 0 |
|  | ≥ 3 food groups/day and  sugary drink ≤ 1 time/week | 1 |
| Dietary diversity | Consumption of < 3 food groups/day | 0 |
|  | Consumption of  ≥ 3 food groups/day | 1 |
| Sleep Duration | < 8 h/day | 0 |
|  | ≥ 8 h/day | 1 |
| Screen Time | 5-18 years:  ≥ 1 h/day  >18 years:  ≥ 2 h/day | 0 |
|  | 5-18 years: < 1 h/day  >18 years: < 2 h/day | 1 |
| Screen Time-S | ≥ 2 h/day | 0 |
|  | < 2 h/day | 1 |
| Physical Activity | < 3 times per week or each session lasting no less than one hour | 0 |
|  | ≥3 times per week with each session lasting no less than one hour | 1 |

^*^ The recommended food groups include vegetables, fruits, whole grains, aquatic products (primarily fish and seafood), and meats.

**Table S2.** Variance inflation factors for covariates in the fully adjusted model

| **Variable** | **VIF** |
| --- | --- |
| HLS | 1.085 |
| Sex | 1.012 |
| Residence | 1.016 |
| Age | 1.192 |
| Father’s employment | 1.411 |
| Mother’s employment | 1.443 |
| Father’s education level | 1.553 |
| Mother’s education level | 1.667 |
| Household income | 1.157 |
| BMI | 1.095 |

**Table S3.** Linear regression models of HLS with PFI and physical fitness outcomes without adjustment for BMI.

|  | **Crude Model** | | | | **Multivariable-adjusted Model ^a^** | | | |
| --- | --- | --- | --- | --- | --- | --- | --- | --- |
|  | **β-coefficient** | **95% CI** | **P-value** | **Adjusted P-value (FDR) ^b^** | **β-coefficient** | **95% CI** | **P-value** | **Adjusted P-value (FDR) ^b^** |
| PFI | 0.47 | (0.43, 0.50) | <0.001* | <0.001* | 0.43 | (0.39, 0.46) | <0.001* | <0.001* |
| Grip strength | 0.03 | (0.02, 0.04) | <0.001* | <0.001* | 0.05 | (0.04, 0.06) | <0.001* | <0.001* |
| 30-s sit-ups | 0.09 | (0.09, 0.10) | <0.001* | <0.001* | 0.09 | (0.08, 0.10) | <0.001* | <0.001* |
| Standing long jump | 0.05 | (0.04, 0.06) | <0.001* | <0.001* | 0.06 | (0.05, 0.07) | <0.001* | <0.001* |
| Sit-and-reach | 0.04 | (0.03, 0.05) | <0.001* | <0.001* | 0.04 | (0.03, 0.04) | <0.001* | <0.001* |
| 50 m dash | -0.06 | (-0.06, -0.05) | <0.001* | <0.001* | -0.07 | (-0.08, -0.06) | <0.001* | <0.001* |
| 20 m SRT | 0.07 | (0.06, 0.08) | <0.001* | <0.001* | 0.08 | (0.07, 0.09) | <0.001* | <0.001* |
| 20-s repeated straddling | 0.03 | (0.02, 0.04) | <0.001* | <0.001* | 0.04 | (0.03, 0.05) | <0.001* | <0.001* |

Abbreviations: CI, confidence interval; PFI, Physical fitness index.

^a^ Adjusted for age, sex (girl or boy), residence (27 different provinces), parental occupation (Civil servant; Worker; Company staff; Business owner (including self-employed); Farmer; Other), parental education (less than primary high school; high school or equivalent; some college or above), household income(less than RMB 2,000; RMB 2,001 to 5,000; RMB 5001-8000; more than RMB 8,000).

^b^ P-values were adjusted for multiple comparisons using the Benjamini-Hochberg false discovery rate (FDR) procedure.

^*^ The results were statistically different, P <0.05.
